# A striking reduction of simple loudness adaptation in autism

**DOI:** 10.1038/srep16157

**Published:** 2015-11-05

**Authors:** Rebecca P. Lawson, Jessica Aylward, Sarah White, Geraint Rees

**Affiliations:** 1 UCL Institute of Cognitive Neuroscience, Alexandra House, 17 Queen Square, London WC1N 3AR; 2 Wellcome Trust Centre for Neuroimaging, University College London, 12 Queen Square, London, WC1N 3BG.

## Abstract

Reports of sensory disturbance, such as loudness sensitivity or sound intolerance, are ubiquitous in Autism Spectrum Disorder (ASD) but a mechanistic explanation for these perceptual differences is lacking. Here we tested adaptation to loudness, a process that regulates incoming sensory input, in adults with ASD and matched controls. Simple loudness adaptation (SLA) is a fundamental adaptive process that reduces the subjective loudness of quiet steady-state sounds in the environment over time, whereas induced loudness adaptation (ILA) is a means of generating a reduction in the perceived volume of louder sounds. ASD participants showed a striking reduction in magnitude and rate of SLA relative to age and ability-matched typical adults, but in contrast ILA remained intact. Furthermore, rate of SLA predicted sensory sensitivity coping strategies in the ASD group. These results provide the first evidence that compromised neural mechanisms governing fundamental adaptive processes might account for sound sensitivity in ASD.

ASD is a neurodevelopmental condition characterized by both social-communicative difficulties and stereotyped or repetitive behavior. In the revised diagnostic criteria for autism, the DSM-5, hyper- or hyposensitivity to sensory stimuli is mentioned as part of the non-social behavioural symptoms of the disorder^1^, and sensory disturbance has an estimated prevalence of ~90% in diagnosed individuals^2^. In explaining sensory perception in ASD, popular theories have focussed on imbalances between local and global sensory processing, most prominently the weak central coherence hypothesis (WCC)^3^, or superior low-level perceptual functioning, Enhanced Perceptual Functioning (EPF) theory^4^. More recent evidence has moved the field forward in terms of a mechanistic understanding of what might underlie these cognitive difficulties and suggests that altered sensory adaptation may contribute to atypical facial processing in ASD^5^,^6^, and is hypothesised to relate to a reduced neural sensitivity to the prevailing sensory context in ASD^7^,^8^,^9^.

Adaptation refers to the change in neural and behavioral responses that accompany prolonged exposure to an adapting stimulus. It is pervasive in sensory systems and one suggestion is that adaptation reduces redundancy by biasing perception away from repeated features in the environment, making novel stimuli and features more salient^10^. Adaptation is thus a fundamental means of using recent spatial or temporal sensory context to inform current perception. Crucially, if adaptive processes did not function within an individual, perceptual constancy would be compromised. Autistic children show reduced adaptation to high-level representations of social cues, like facial identity^5^. This may have important implications for understanding the social impairments that are prevalent in autism. However, beyond social stimuli adaptive processes are constantly shaping the way we experience the world. This raises the intriguing possibility that sensory sensitivity reported in autism could result from compromised adaptive processes in perceptual-sensory domains^9^.

In the auditory domain, for example, loudness adaptation putatively ‘re-calibrates’ our auditory sensitivity for the continuous sounds in our environment^11^, like the hum of an air conditioning unit, making loud or changing sounds more salient. In this regard adaptation is a mechanism to adjust the gain (or precision) of sensory inputs, a process that has been proposed to be aberrant in ASD^1^. If adaptive processes governing the perception of loudness were compromised in ASD this could offer a mechanistic explanation for increased loudness sensitivity which is often reported^12^,^13^. Reduced adaptation to sensory stimuli might predict overall sensory sensitivity, or management of sensory symptoms, in adults with ASD. To date, however, no studies have investigated the integrity of such fundamental adaptive processes, or adaptation in the auditory domain, in ASD.

Unlike other sensations, loudness is surprisingly stable over time. Pioneering work in the 1980’s revealed two special conditions in which our perception of loudness decreases as a function of continued stimulation^14^. *Simple* loudness adaptation (SLA) is the subjective decrease in loudness that occurs over time when a quiet and continuous pure tone, typically below 40 dB SPL, is played monaurally^15^. If SLA were compromised in autism, this might offer mechanistic explanation for why quiet sounds that most people would consider innocuous can be extremely disturbing to people on the autism spectrum. In contrast, *induced* loudness adaptation (ILA) is a means of reducing the perceived volume of louder tones, typically above 50 Db SPL, as the result of an intermittent tone presented to the contralateral ear^15^. If ILA were compromised in autism, this might suggest that interaural context does not constrain the volume of loud sounds. ILA and SLA, then, provide a means to explore adaptation to loud and quiet tones respectively.

Here we tested SLA and ILA in twenty adults with a diagnosis of ASD (12 male) and twenty neurotypical controls (10 male), matched for age and IQ. Group demographics and questionnaire scores can be seen in Table 1. In the SLA condition a continuous 1 kHz tone (30 dB SPL) was played monaurally to the right ear for a total duration of 190 seconds (Fig. 1B). In the ILA condition a 1 kHz tone (70 dB SPL) was played to the right ear for 190 seconds and an intermittent 1 kHz tone (75 dB SPL) was played to the contralateral left ear (Fig. 1A). In both tasks participants made periodic ratings of loudness, from which measures of adaptation magnitude and rate were calculated in accordance with previous research^15^,^16^.

## Results

To examine differences in adaptation magnitude we conducted a repeated-measures ANOVA with a within-subject factor of task (ILA, SLA) and a between-subject factor of group (ASD, control). Even though both groups were matched on age, we included age and also sex as covariates in this analysis to control for the higher than usual number of females and also any general age-related hearing abilities across the sample. This analysis demonstrated that the ASD group adapted significantly less than the control group on average across both tasks (F(1, 36 = 4.73, p = 0.036, ηp^2^ = 0.12). This group difference was qualified by a significant task*group interaction (F(1, 36) = 6.57, p = 0.015, ηp^2^ = 0.15), indicating that the difference in magnitude of adaptation between the groups differed between the two tasks. There was no interaction of task with age (F(1, 36) = 0.47, p = 0.5, ηp^2^ = 0.01), or sex (F(1, 36) = 1.16, p = 0.29, ηp^2^ = 0.03).

An equivalent analysis of adaptation rate found a main effect of group (F(1, 36) = 4.86, p  =  0.034, ηp^2^ = 0.12), meaning that, on average across both tasks, the rate of adaptation was slower for the ASD group than the control group. A significant group*task interaction (F(1, 36) = 5.83, p = 0.021, ηp^2^ = 0.14) demonstrated that the difference in the rate of adaptation between the groups differed between the two tasks. Again, there was no interaction with age (F(1, 36) = 0.57, p  =  0.45, ηp^2^ = 0.02) or sex (F(1, 36) = 3.62, p = 0.07, ηp^2^ = 0.09).

Posthoc t-tests showed that in the SLA task there was a striking reduction in adaptation magnitude in the ASD group relative to controls (t(38) = 3.70, p = 0.001). The rate of adaptation was also slower for the ASD group versus controls (t(38) = 3.22, p = 0.003; Fig. 2A). In the ILA task, however, neither magnitude (t(38) = 0.47, p = 0.64) nor rate of adaptation (t(38) = −0.47, p = 0.64) differed between the groups (Fig. 2B).

To test whether compromised SLA might relate to self-report sensory symptoms we conducted bivariate correlations between ASQ and the rate and magnitude of SLA. While adaptation rate and magnitude did not correlate with overall sensory sensitivity scores in the ASD group (rate: *r*(20) = 0.215, *p* = 0.182; magnitude; *r*(20) = 0.021, *p* = 0.47), the ASD participants who scored highly on the ‘coping strategies’ subscale had faster rates of SLA (steeper slopes)(*r*(20) = 0.44, *p* = 0.026; Fig. 2C). There was no correlation between ‘coping strategies’ and SLA rate in the controls (*r*(19) = −0.16 , *p* = 0.22; Fig. 2D). This suggests that adults with ASD who employ coping strategies to manage their sensory stress show SLA rates that are more like controls.

## Discussion

Our findings suggest that adaptation to the loudness of quiet steady-state sounds (SLA) is compromised in ASD, whereas adaptation to loud steady-state sounds (ILA) does not differ from controls. This could offer a mechanistic explanation, i.e. a failure of adaptive coding mechanisms that regulate incoming sensory input, for the symptoms of loudness sensitivity and sensory ‘overload’ often reported in ASD. Notably, however, we found no relationship between overall measures of sensory sensitivity and adaptation rate or magnitude whereas, in the ASD participants, SLA rate was predicted by tendency to employ coping strategies to manage sensory input.

It is interesting to consider why only SLA and not ILA is compromised. Many studies suggest a different neural basis for these two processes. Studies probing cognitive factors influencing SLA suggest it is a sensory phenomenon (rather than response bias) likely to arise from the central auditory system^17^. Neurosurgical patients with severed olivocochlear bundles exhibit intact SLA, whereas ILA is heterogeneously impaired^16^, strong evidence of distinct neural substrates. ILA has been linked to the phenomenon of induced loudness reduction (ILR) and it has been proposed that they may share a common physiological basis^18^, which may be interaural interactions under binaural conditions^15^. The difficulty in producing ILA under normal listening conditions might reflect the fact that it’s not advantageous to habituate to salient loud sounds, such as alarms, which ought to grab and sustain our attention. In ASD, however, a failure to adapt adequately to quiet steady-state sounds would result in awareness of these sounds persisting over time and could offer a mechanistic explanation for *why* sounds that most people would consider innocuous, or mildly bothersome, can be extremely disturbing to people on the autism spectrum (i.e. a failure of SLA). This link between mechanism (adaptation) and symptom (perceived loudness of sounds) goes beyond what we already know, namely that people with ASD can be less tolerant of certain sounds^19^. We acknowledge, however, that the details of these mechanisms (SLA, ILA) are not well understood in normal listeners. Nevertheless our finding that ILA, but not SLA, is preserved in autism lends credence to the hypothesis that they have different neural substrates. It is, however, beyond the scope of the current paper to resolve what these different neural substrates are.

It has recently been questioned whether certain “high-level” visual aftereffects reflect genuine perceptual changes or non-perceptual shifts in a participant’s decision criterion^20^. This issue mostly applies to forced-choice classification of stimuli during method-of-single-stimulus tasks. This study, however, employs successive magnitude estimation (a variant of Steven’s magnitude estimation^21^). In allowing participants to make a parametrically varying response on every trial (not a forced choice decision), we circumvent simple biases in an individual’s decision criterion to, for example “always report quieter”. Participants have to judge *how much* quieter they believe the stimulus to now be and ascribe this percept a numerical value. We know, from the fact that both groups adapt the same amount in the ILA task, that the SLA results cannot easily be explained by group differences in task strategy (e.g. a general bias to report no change) or understanding – which would have affected both tasks equally. Indeed, the present study goes beyond the previously reported differences in perceptual adaptation *aftereffects* because the effects measured here are not aftereffects *per se*. Loudness adaptation relates to changes in estimates of sensation over time as a function of continuous stimulation; like habituation. It is not presently clear how adaptation in this sense relates formally to perceptual aftereffects in high- and low-level visual domains, which seem impaired^5^,^6^,^22^,^23^ and preserved^24^ respectively, in children with ASD. Clearly more research is needed, including studies of sensory adaptation in other domains where perception changes gradually over time–such as light and dark adaptation.

Atypical central auditory processing in ASD occurs without peripheral hearing problems^25^ suggesting that auditory perceptual differences in this population do not simply result from abnormal inner ear morphology. Although we did not conduct hearing tests or acquire auditory thresholds for our participants we ensured that members of both groups had normal hearing, no history of hearing problems and could hear the initial stimuli presented to them in both tasks. This is similar to the requirements for a typical visual perception study. Therefore, group differences in the peripheral auditory system are unlikely to explain our results. Furthermore we included age (as a proxy for general age-related hearing abilities) as covariates in all analyses. Instead, it seems likely, that the problems stem either from an abnormality of primary auditory cortices or atypical, top-down control over primary auditory or even inner ear structures^26^. Neurobiologically, an imbalance of the precision (or neuromodulatory gain) ascribed to sensory inputs, relative to the top-down predictions of those inputs, has been hypothesized as a recent account of ASD^7^,^8^,^27^. Under this scheme a failure of sensory attenuation is one mechanism proposed to underlie this aberrant balance of precision^8^. Top-down (feedback) mechanisms have been implicated in fMRI-adaptation/repetition suppression to visual stimuli^28^ and reduced fMRI-adaptation is observed in ASD^29^, schizophrenia^30^ and also correlates with individual differences in autistic traits^31^. Future neuroimaging studies of auditory adaptation in ASD may begin to explain the etiology of sensory processing difficulties in the disorder and may help dissociate WCC from EPF theories.

We found that the rate of SLA in ASD individuals was predicted by self-report tendency to employ coping strategies to deal with sensory sensitivity; ASD participants who adapt faster are more likely to successfully employ coping strategies to deal with sensory stress. This raises the important issue of compensatory mechanisms when exploring perceptual (or even social) task performance in adults with ASD. Within the visual domain, adults with ASD show comparable facial aftereffects to neurotypical controls^32^ in contrast to earlier reports of reduced facial identity aftereffects in children with ASD^5^. Compensatory mechanisms that are acquired throughout development might account for this discrepancy 32. Here we *do* find a group difference in the magnitude and rate of SLA in an adult sample. This difference between studies could be due to differences in the modality of study (auditory vs. visual) and also differences in the level of processing under study (simple pure tones vs. high level faces). Additionally, the correlation with ‘coping strategies’ in the ASD group suggests that the ASD participants who adapt fastest to quiet steady-state sounds are those who most often employ active strategies to manage sensory stress (i.e. mentally preparing for situations when sensory stress is unavoidable). It is possible that learned coping strategies across one’s lifetime entrain better regulation of sensory input, and our data provide encouraging preliminary evidence for this. Interestingly, ‘coping strategies’ only predicted the rate of SLA, and not the overall magnitude of SLA, which suggests that the use of active coping strategies in ASD may help in adapting faster to sounds, but do not necessarily affect how much adaptation will take place overall. Future research including longitudinal studies and controlled trials of different sensory coping methods are necessary to further explore this hypothesis.

Sensory processing in autism is an area of increasingly intense investigation, largely because sensory symptoms are among some of the most challenging. Our findings offer new insight into these difficulties, highlighting the role of abnormal adaptive mechanisms processing fundamental auditory stimuli and demonstrating a preliminary relationship between loudness processing and sensory coping strategies. It is worth noting, however, that this difference in the extent to which people with autism can regulate their perception of quiet and continuous sounds can’t be viewed as straightforwardly disadvantageous – the tone doesn’t actually change in volume and the autistic group are perceiving the world more veridically in this respect. Whether these differences in the ability to regulate incoming sensory input extends to complex auditory stimuli or are related to the sensory adaptation in other domains warrants further investigation.

## Materials and Methods

### Participants

Twenty participants (12 male) diagnosed with ASD were recruited via the Autism database held at the UCL Institute of Cognitive Neuroscience. Twenty participants (10 male) with no previous or current psychiatric diagnosis served as controls. ASD participants had previously been diagnosed by an independent clinician, according to the DSM-IV^33^ or ICD-10^34^ criteria [17 Asperger Syndrome, 2 Autistic Disorder, 1 High Functioning Autism]. No participants were excluded or dropped out. ASD participants were also tested on the Autism Diagnostic Observation Scale (ADOS-G^35^) to verify diagnoses. This data was unavailable for five of the ASD participants who were not excluded as they had Autism Quotient (AQ) scores close to or above the cut off^36^ and results were not altered by exclusion of these participants. The Wechsler Adult Intelligence Scale (WAIS 3^rd^ edition UK) had previously been administered to assess IQ. For six of the participants IQ was assessed using the Wechsler Abbreviated Scale of Intelligence, second edition (WASI-II^37^. Group demographics and questionnaire scores can be seen in Table 1. The Adult Sensory Questionnaire (ASQ) is a self-report measure of sensory defensiveness which provides an overall score (larger numbers indicate greater sensory defensiveness) and also can be broken down into sub-measures of general sensitivity to, emotional regulation of and coping strategies for, sensory stimulation^38^.

Ethical approval was provided by the UCL Research Ethics Committee (4357/001) and all methods were carried out in accordance with the approved guidelines. All participants were provided with verbal task instructions and were given an information sheet to read prior to providing written informed consent. All participants self-reported no vision or hearing problems and were reimbursed for their time, at a rate of £7.50 per hour.

### Stimuli

Stimuli were generated digitally at a sampling rate of 48,000 kHz using MATLAB 7.7.0471 (R2008b) (http://www.mathworks.co.uk/) and presented with Cogent 2000 (http://www.vislab.ucl.ac.uk/cogent_2000) on a Dell Precision M4500 laptop. Sounds were 1000 Hz pure tones gated on and off with cosine ramps presented via Sennheiser 201 headphones. Tone volume was measured with a Brüel & Kjær (B&K) artificial ear type 4153 fitted with a B&K 4192 half inch condenser microphone and a B&K 2669 preamplifier. An adapter plate was fitted to the artificial ear to insure a good acoustic seal when testing circumaural headphones. The signal was captured with a B&K PHOTON+ which was connected to a laptop running RTPro 7.2 signal analysis software. This was calibrated with a B&K 4220 Pistonphone −250 Hz sinewave at a nominal RMS intensity of 124 dB SPL.

### Procedure

Participants were informed that they were required to make a series of loudness judgements of a tone presented to their right ear. As in previous studies of loudness adaptation, perceived loudness was measured using successive magnitude estimation which is as reliable as other procedures^39^. Methods for testing loudness adaptation closely followed previous research in typical listeners^15^. A fixed number of ‘100’ represented the initial volume of the tone for all participants in both ILA and SLA conditions. Imposing this initial loudness value controlled for any differences between the groups in the creation, and use, of the volume metric. Before proceeding it was checked that every participant could actually hear the tone. Participants were told that the tone “might change in volume”. At fixed intervals participants rated the loudness of the tone in their right ear relative to the initial loudness and wrote their responses down on a provided response sheet following a visual prompt on screen to do so. Participants were instructed to use the range of numbers “as if they represented the volume of a television or radio”. Relative to the initial volume, a rating of ‘50’ should be given if the volume decreased by half, a rating of ‘150’ should be given if the volume increased by a half, and rating of ‘100’ would mean the volume hadn’t changed. A rating of ‘0’ should be given any time they could no longer hear the tone. The adaptation tasks (ILA and SLA) were presented in a counterbalanced order.

### Induced Loudness Adaptation (ILA)

**Figure 1:**
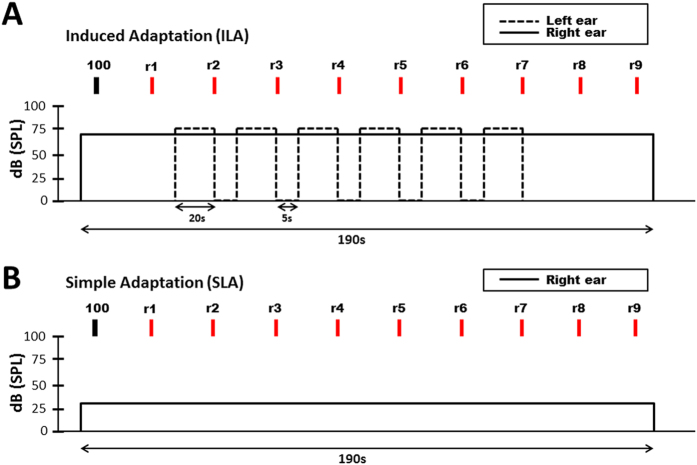
Loudness adaptation tasks. (**A**) Shows the timeline for Simple Loudness Adaptation (SLA) and (**B**) for Induced Loudness Adaptation (ILA). Solid line shows pure tone (in dB SPL ) being presented to the right ear. Dotted line shows pure tone (in dB SPL) being presented to the left ear. R1 = loudness rating 1, R2 = loudness rating 2 etc. See main text for more details.

**Figure 2:**
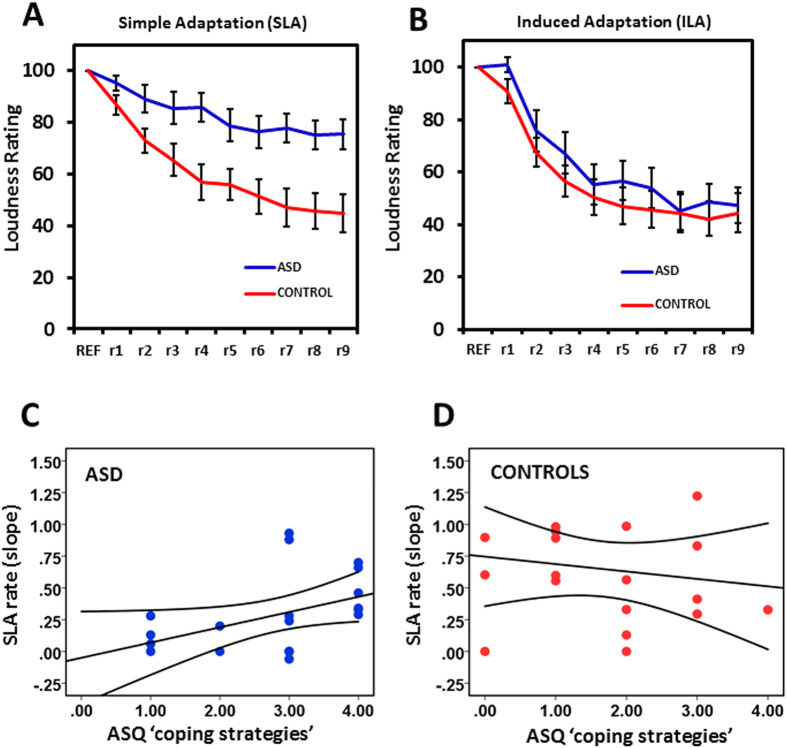
Loudness adaptation results. (**A**) shows the results for the SLA task. Rate and magnitude of loudness adaptation was significantly reduced for the ASD group (blue) relative to the control group (red) (**B**) ASD (blue) and control (red) groups did not differ on rate and magnitude of adaptation in the ILA task. (**C**) shows a significant correlation between SLA rate and self-report ‘coping strategies’ in the ASD group, whereas (**D**) this correlation was not present in the controls. Data are represented as mean +/− SEM.

A 70 dB SPL tone was presented continuously to the right ear for 190 seconds. After 20 seconds (during which participants heard only the tone in the right ear and were given the volume rating for this tone i.e. 100), an 75 dB SPL tone was presented to the left ear intermittently (15 seconds on, 5 seconds off) and then switched off after 120 seconds, after which time only the tone in the right ear remained. Participants rated right ear loudness every 20 seconds, with the first rating given after fifteen seconds following a visual cue (“rate right ear loudness now”). Participants made nine ratings in total; the final two were made after the intermittent tone was switched off (Fig. 1A).

### Simple Loudness Adaptation (SLA)

A 30 dB SPL tone was presented continuously to the right ear for 190 seconds. After 20 seconds (in which participants were given the volume rating for this tone i.e. 100), participants rated loudness every 20 seconds in exactly the same manner as for ILA adaptation above. Participants made nine ratings in total. No tone was presented to the left ear (Fig. 1B).

### Adaptation rate and magnitude

For SLA, adaptation magnitude was calculated by subtracting the average of the final two loudness estimates from the initial volume rating of ‘100’ − equivalent to calculating a percentage change. For ILA, the average of the final two ratings made in the presence of the intermittent ‘adapting’ tone (the 6^th^ and 7^th)^ were subtracted from the initial rating of 100. Adaptation rate was calculated by fitting a hyperbolic decay function to the per-participant successive loudness ratings and the resultant beta coefficients were used as an index of how quickly the loudness was perceived to change.

## Additional Information

**How to cite this article**: Lawson, R. P. *et al.* A striking reduction of simple loudness adaptation in autism. *Sci. Rep.* **5**, 16157; doi: 10.1038/srep16157 (2015).

## Figures and Tables

**Table 1:** Participant demographics and questionnaire scores.

Measure	Group				*t*	*df*	*P*	
	ASD		control					
	mean (sd)	range	mean (sd)	range				
Age (years)	41.45(11.61)	27–68	40.30(11.59)	21–59	0.31	38.00	0.77	
Full scale (IQ)	112.25(17.66)	80–144	111.35(13.00)	82–127	0.18	38.00	0.86	
ADOS–total	8.73(0.72)	4–17	n/a	n/a				
AQ	36.25(9.55)	17–48	16.35(5.20)	8–26	8.18	29.37	<0.001*	
ASQ†								
sensory sensitivity	5.80(0.61)	2–11	4.05(0.56)	0–9	2.07	37.00	0.045*	
soc–emot behaviour	2.60(0.26)	0–4	0.68(0.17)	0–2	6.15	37.00	<0.001**	
self-regulation	2.60(0.31)	0–4	1.7(0.27)	0–4	2.23	37.00	0.032*	
coping strategies	2.80(0.25)	0–4	1.21(0.29)	0–3	4.17	37.00	<0.001**	
total	13.80(4.24)	6–20	7.75(3.63)	2–14	4.85	38.00	<0.001**	

^†^Subscale data missing for one control.

^**^p < 0.001, *p < 0.05.
